# A Graph-Based Ant Colony Optimization Approach for Process Planning

**DOI:** 10.1155/2014/271895

**Published:** 2014-06-03

**Authors:** JinFeng Wang, XiaoLiang Fan, Shuting Wan

**Affiliations:** School of Energy, Power and Mechanical Engineering, North China Electric Power University, Baoding 071003, China

## Abstract

The complex process planning problem is modeled as a combinatorial optimization problem with constraints in this paper. An ant colony optimization (ACO) approach has been developed to deal with process planning problem by simultaneously considering activities such as sequencing operations, selecting manufacturing resources, and determining setup plans to achieve the optimal process plan. A weighted directed graph is conducted to describe the operations, precedence constraints between operations, and the possible visited path between operation nodes. A representation of process plan is described based on the weighted directed graph. Ant colony goes through the necessary nodes on the graph to achieve the optimal solution with the objective of minimizing total production costs (TPC). Two cases have been carried out to study the influence of various parameters of ACO on the system performance. Extensive comparative experiments have been conducted to demonstrate the feasibility and efficiency of the proposed approach.

## 1. Introduction

Process planning is the function which translates the design requirements into the detailed technologically feasible instructions, which involves selecting machining operations, sequencing these operations, choosing manufacturing resources, determining setup plans, machining parameters, and so forth. These activities must be carried out simultaneously to achieve an optimal process plan. But, due to the complexity of part structures and variability of machining environment, process planning is well known as a complicated decision-making process. Computer-aided process planning (CAPP) system will assist human planners in completing the process planning, which is an essential component for linking the various models and processes in a computer-integrated manufacturing system (CIMS) [1].

With the development of computer technologies, CAPP has received much attention during the last three decades and played an increasingly important role in a CIMS [2]. The initial “variant” CAPP systems are based on the group technology (GT) coding and classification system. A baseline process plan for a part family has been defined in such systems. According to the part code, approximately 90% of the process plans can be yielded automatically while the remaining 10% is achieved through modifying the process plans manually. The application of artificial intelligence in CAPP system accelerates the generation of a complete process plan, namely, from the variant CAPP system to the generative CAPP system. A generative CAPP system consists of three main consecutive activities: (1) identifying manufacturing features, (2) determining feasible machining operation and available machining resources, and (3) selecting machining operation and machining resources and sequencing machining operations [3, 4]. This paper focuses on the solution of the third activity and presents an ACO approach to solve the process planning problem.

The rest of this paper is organized as follows.  Section 2 introduces previous related work. Process planning problem is described in Section 3. The proposed ACO approach for process planning is given in Section 4. In Section 5, simulation experiments are made and the results are discussed compared with other approaches. Finally, Section 6 concludes the present study and outlines the future study.

## 2. Previous Related Works

In the past three decades, many optimization approaches have been developed and widely applied for solving process planning problem, such as knowledge-based reasoning approach [5, 6], graph manipulation [7, 8], the genetic algorithm (GA) [9–11], tabu search approach (TS) [4, 12], simulated annealing (SA) algorithm [3, 13], particle swarm optimization (PSO) [14, 15], artificial neural networks [16], ant colony optimization (ACO) [17, 18], and artificial immune system (AIS) [19].

Usher and Sharma [5] proposed an approach of intelligent reasoning based on the feathers of part. Many constraints and criteria were present in operation planning, which were analyzed intelligently to generate the potential operation plans. Usher and Bowden [1] apply an improved operation sequence coding of genetic algorithms for process planning problem, which can determine optimal operation sequences for parts of varying complexity. Zhang et al. [10] proposed a GA for a novel computer-aided process planning (CAPP) model in a job shop manufacturing environment. GA is used to select machining resources and sequence operations simultaneously. The dynamic status of machining resources in the job shop and alternative optimal plans are not taken into account. Li et al. [4] consider the process planning problem as a constraint-based optimization problem and propose a tabu search-based approach to solve it. In the proposed optimization approach, precedence constraints between features and their related operations are defined and classified according to their effects on the plan feasibility and processing quality. Ma et al. [13] modeled the constraints of process planning problems in a concurrent manner. Precedence relationships among all the operations are used to generate the entire solution space with multiple planning tasks. Based on the proposed model, they used an algorithm based on simulated annealing (SA) to search for the optimal solution. Guo et al. [14] proposed a PSO approach to operation planning problem. The initial process plans randomly generated are encoded into particles of the PSO algorithm. To avoid falling into local optimal and improve the particles' movements, several new operators have been developed. Penalty strategy is used considering the evaluation of infeasible particles. Krishna and Mallikarjuna Rao [17] proposed a novel approach to apply the ant colony algorithm as a global search technique for process planning problem by considering various feasibility constraints. Chan et al. model the machine tool selection and operation allocation of flexible manufacturing systems and solve process problem by a fuzzy goal—programming approach based on artificial immune systems.

Recently, to improve the quality of results and efficiency of the search, many hybrid approaches are developed for process planning problem, for example, GA + SA [3], graph manipulation + GA [8], and local search algorithm + PSO [20]. Li et al. [3] developed a hybrid genetic algorithm and a simulated annealing approach for optimizing process plans for prismatic parts. They modeled the process planning as a combinatorial optimization problem with constraints. The evaluation criterion was the combination of machine costs, cutting tool costs, machine change costs, tool change, and setup costs. Ding et al. [20] proposed a hybrid approach to incorporate a genetic algorithm, neural network, and analytical hierarchical process (AHP) for process planning problem. A globally optimized fitness function is defined including the evaluation of manufacturing rules using AHP, calculation of cost and time, and determination of relative weights using neural network techniques. Huang et al. [8] model the process planning problem as a combinatorial optimization problem with constraints and developed a hybrid graph and genetic algorithm (GA) approach. In the approach, graph theory accompanied with matrix theory is embedded into the main frame of GA. The precedence constraints between operations are formulated in an operation precedence graph (OPG). An improved GA was applied to solve process planning problem based on the operation precedence graph (OPG). Wang et al. [21] proposed an optimization approach based on particle swarm optimization (PSO) to solve the process planning problem and introduced a novel solution representation scheme for the application of PSO. In the hybrid approach, two kinds of local search algorithms are incorporated and interweaved with PSO evolution to improve the best solution in each generation.

Although significant improvements have been achieved for process planning problem, there still remains potential for further improvement [22]. For example, optimization approach needs to be improved to be more efficient, and a more reasonable constraint modeling and handling mechanism needs to be developed; also, some practical manufacturing environment should be considered, and the approach should provide the multiple alternative optimal plans.

## 3. Process Planning Problem Description

### 3.1. Process Plan Representation

In CAD systems, a part is generally described by features with specific machining meanings, such as planes, chamfers, holes, slots, and steps. Given a part and a set of manufacturing resources, the process planning problem of CAPP can be described as follows.

**Figure 1:**
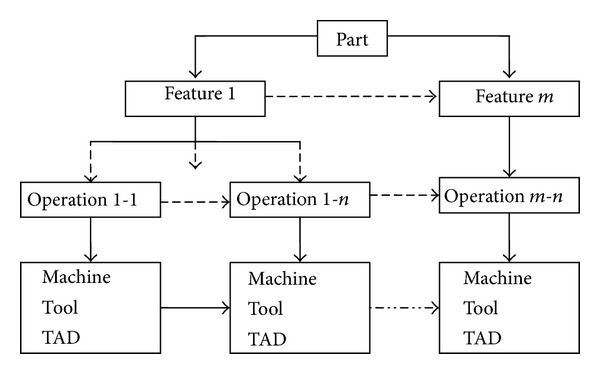
The representation of process plan for a part with *m* feathers.

The CAD information of part is read before process planning. Then, the machining method of each feature is selected according to the attributes of different features, which can be expressed by the various operations eventually. So, it is necessary to determine one or several operations for each feature in advance. The operations consist of machines, cutting tools, and tool approach directions (TAD). A TAD is defined as a direction from which a cutting tool can access a feature [7, 10]. For each feature of part, the selection of machines, cutting tools, and TADs is based on the feature geometry and available machining resources. For a part with *m* feathers, the relationships between part, feather, and operation are shown in Figure 1.

**Figure 2:**
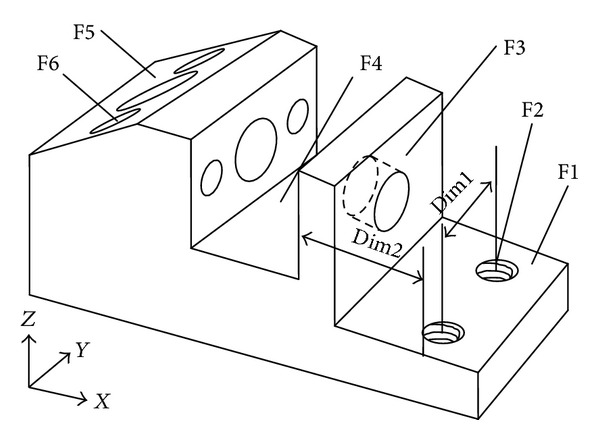
An example part.

An example part is shown in Figure 2. The part includes six feathers: F1 (a step), F2 (two holes arranged in a replicated feature), F3 (a through hole), F4 (a slot), F5 (a chamfer), and F6 (two blind holes arranged in a replicated feature). Some feathers may have more than one machining method. Each machining method has different selection of machines, cutting tools, and TADs. For the example part, the feather of F3 may have two different machining methods of drilling→reaming and drilling→grinding. However, it is possible to have different combination of machines, cutting tools, and TAD even though the selection is overlapped [11]. For any part, TAD includes six directions, that is, +*X*, −*X*, +*Y*, −*Y*, +*Z*, and −*Z*. However, it is common that some TAD will be likely to be discarded for the interference between feathers. For example, the drilling of F6 has two possible TADs, that is, −*X* and +*X*, because the tool cannot access the hole from the direction of −*X*, and the TAD of −*X* will be discarded. The features and their valid TADs can be recognized using a geometric reasoning approach [23, 24]. The final result of operation selection for the example part is shown in Table 1. “Op” represents operation; for example, “Op_1_” represents operation 1. There is only 1 operation for the feathers of F1, F4, and F5 and 2 operations for the feathers of F2, F3, and F6.

### 3.2. Precedence Constraints

Process planning involves determining in what order to perform a set of selected operations such that the resulting order satisfies the precedence constraints. These constraints are established by considering both a large number of geometrical interactions and technological requirements between the various factors [1, 3, 8, 25], which cause process planning to become more complicated. The constraints can be divided into the feasibility constraints and optimality constraints [1]. A feasible process plan is deemed to be one which does not violate any of the feasibility constraints. The optimality constraints affect the quality, cost, and efficiency of a feasible process plan, which may be violated at certain times in cases of contradictions to some feasibility constraints. Faheem et al. indicate constraint affecting the generation of process plans which can be classified as “hard” or “soft” constraints [25]. Hard constraints affect the manufacturing feasibility and a process plan should be consistent with these constraints. Soft constraints only affect the quality, cost, or efficiency of a feasible process plan. Many constraints and rules have been proposed and summarized [1, 4, 9, 10]. These precedence constraints are summarized as follows [18].

**Rule 1**

Primary surfaces prior to secondary surface.

**Rule 2**

Planes prior to its associated features.

**Rule 3**

Rough machining operation prior to finishing machining operation.

**Rule 4**

Datum surfaces prior to its associated features.

**Rule 5**

Some good manufacturing practice. For example, features related to thin wall should be machined first; features that caused tool damage and failure of clamping potentially should be machined before or later, and feathers that affect the cost or the quality of machining should be machined first.

These constraints between machining operations can be used to constrain the search in a smaller space and enhance search efficiency. Some examples of the above precedence constraints for the example part in Figure 2 are illustrated in Table 2.

### 3.3. Process Plan Evaluation Criterion

The most common evaluation criteria for process plan include minimum number of setups, shortest process time, and minimum machining cost. Váncza and Márkus used number of setups, number of tool changes, and total cost of individual operations as evaluation criteria [9]. Usher and Sharma used number of setups, continuity of motion, and loose precedence as evaluation criteria [5]. Zhang et al. used machine costs, cutting tool costs, number of machine changes, number of tool changes, and number of setups as evaluation criteria [10]. Many evaluation criteria have been proposed, which include process time, number of setups, number of tool changes, number of machine changes, continuity of motion, and total cost of individual operations. Because the detailed information on machining parameters is not available at this stage, the total machining time cannot be used for plan evaluation. In this paper, five cost evaluation criteria are adopted and are similar to the criteria in paper [3, 4].

(1) Total machine cost (TMC) is
  (1) $$\begin{array}{c} \text{TMC}=\overset{n}{\underset{i=1}{\sum }}\text{M}{\text{C}}_{i}, \end{array}$$
  where *n* is the total number of operations and MC_*i*_ is the machine cost of the *i*th machine for an operation, a constant for a specific machine.
(2) Total tool cost (TTC) is
  (2) $$\begin{array}{c} \text{TTC}=\overset{n}{\underset{i=1}{\sum }}\text{T}{\text{C}}_{i}, \end{array}$$
  where TC_*i*_ is the tool cost of the *i*th tool for an operation, a constant for a specific tool.
(3) Total machine change cost (TMCC): a machine change is needed when two adjacent operations are executed on different machines
  (3) $$\begin{array}{c} \text{TMCC}=\text{MCC}*\text{NMC}, \end{array}$$
  where MCC is the machine change cost and NMC is the number of machine changes, which can be calculated by
  (4) $$\begin{array}{c} \text{NMC}=\overset{n-1}{\underset{i=1}{\sum }}\Omega_{1}\left(M_{i+1},M_{i}\right), \end{array}$$
  where *M*_*i*_ is the machine for the *i*th operation and *Ω*_1_(*x*, *y*) is a judging function:
  (5) $$\begin{array}{c} \Omega_{1}\left(x,y\right)=\{\begin{array}{cc} 1 & x\neq y,\\ 0 & x=y. \end{array} \end{array}$$
(4) Total tool change cost (TTCC): a tool change is defined in Table 3 [3]
  (6) $$\begin{array}{c} \text{TTCC}=\text{TCC}*\text{NTC}, \end{array}$$
  where TCC is the tool change cost and NTC is the number of tool changes, which can be calculated by
  (7) $$\begin{array}{c} \text{NTC}=\overset{n-1}{\underset{i=1}{\sum }}\Omega_{2}\left(\Omega_{1}\left(M_{i+1},M_{i}\right),\Omega_{1}\left(T_{i+1},T_{i}\right)\right), \end{array}$$
  where *T*_*i*_ is the *i*th tool. *Ω*_2_(*x*, *y*) is a judging function:
  (8) $$\begin{array}{c} \Omega_{2}\left(x,y\right)=\{\begin{array}{cc} 0 & x=y=0,\\ 1 & \text{otherwise}. \end{array} \end{array}$$
(5) Total setup cost (TSCC): a setup change is defined in Table 4 [3]
  (9) $$\begin{array}{c} \text{TSCC}=\text{SCC}*\text{NSC}, \end{array}$$
  where SCC is the setup cost and NSC is the number of setups, which can be calculated by
  (10) $$\begin{array}{c} \text{NSC}=\overset{n-1}{\underset{i=1}{\sum }}\Omega_{2}\left(\Omega_{1}\left(M_{i+1},M_{i}\right),\Omega_{1}\left(\text{TA}{\text{D}}_{i+1},\text{TA}{{\text{D}}_{i}}_{i}\right)\right)+1, \end{array}$$
  where TAD_*i*_ is the *i*th TAD.
(6) Total production cost (TPC) is
  (11) TPC=w1∗TMC+w2∗TTC+w3∗TMCC+w4∗TTCC+w5∗TSCC.

In (11), TPC is total production cost. *w*_1_, *w*_2_, *w*_3_, *w*_4_, and *w*_5_ are weights of TMC, TTC, TMCC, TTCC, andTSCC, respectively. These weights can be assigned referring to the active situations, which provide the flexibility to customize the optimization objective function according to various situations. The different values of *w*_1_, *w*_2_, *w*_3_, *w*_4_, and *w*_5_ constitute the flexible combination to meet the requirement of process planning in different manufacturing environment. The detailed method of setting these parameters is given in the subsequent sections.

## 4. The Proposed ACO Algorithm

### 4.1. Graph-Based Representation of Process Plan

The proposed ACO algorithm basically generates solutions by standard ACO procedures [26]. To construct a feasible process plan with the ACO approach, the process planning problem has to be visualized and represented by a weighted directed graph [27].

**Figure 3:**
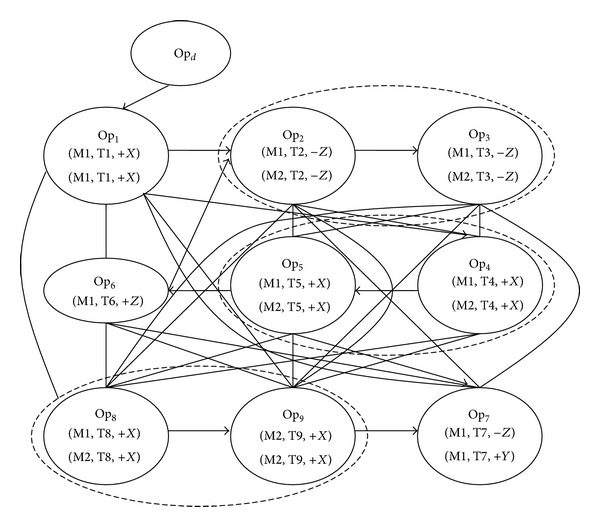
A disjunctive weighted directed graph for the example part.

The weighted graph is denoted by *D* = (*O*, *A*, *B*), where *O* is a set of nodes, *A* is a set of directed arcs, and *B* is a set of undirected arcs. The nodes of *O* stand for all of the operations Op_*i*_, and *A* corresponds to the precedence constraints between the operations of the parts. *B* represents the set of arcs connecting all possible combination of the nodes. Both *A* and *B* represent possible paths for ants travelling from one node to another. The ants are basically free to travel along the paths unless there is a precedence constraint specified by *A*.  Figure 3 is the weighted graph for the example in Figure 2.

The approach in this paper applying the ACO algorithm for process planning is to search for a path in a weighted graph (Figure 3), where all necessary nodes have to be visited to complete the process plan to minimize TPC. The characteristic of this approach is to construct process plans from an autocatalytic process, in which artificial ants favor the process plan with smaller TPC and they will deposit more pheromones on the visited paths so that there is a higher probability for the following ants to continue choosing the better paths.

### 4.2. Initialization

Before starting the ACO for process planning, the ant colony was placed on the initial node. The selection of the initial node determines which features can be machined firstly, which affects the result of process planning and the performance of ACO. Only these operations attached to the features with no precedent features may be selected as the initial node. For the example part in Figure 2, only F1 has no precedent features, so Op_1_ will be allowed to be the first visited node. In fact it is difficult to select the initial node from many operation nodes, because the initial node is not unique in most of the process planning. In this paper a dummy node Op_*d*_, acting as the initial node, is added to the weighted graph to connect the first feasible operations of the parts, as shown in Figure 3. In addition, the undirected arc is added from the initial node to the possibly first visited operation nodes. The number of ants (*K*) in the colony is arbitrary, and it can be set as a parameter, which is allowed to be adjusted in accordance with the scale of the problem and the performance of the algorithm.

### 4.3. Iteration

For the ant *k*, a path will be achieved after traversing all the nodes in a weighted graph, which represents the one of feasible process plans. To choose the next visiting node, the ant *k* is guided by the heuristic information *η*_*uv*_ on the node and the pheromone amount *τ*_*uv*_ on the arc linking the source node *u* and possible destination node *v*. The heuristic information *η*_*uv*_ can reflect the attractiveness of the next visiting node for the ant *k*. When minimizing TPC is used to be objective function for process planning, MC and TC of the operation node will be treated to calculate *η*_*uv*_. The heuristic information  *η*_*uv*_ can be given as follows:

(12) $$\begin{array}{c} \eta_{uv}=\frac{E}{\text{PC}}, \end{array}$$

where *E* is a positive constant, and it can be set by trial and error. PC is the processing cost of the selected node operation and it is calculated as follows:

(13) $$\begin{array}{c} \text{PC}=w_{1}*\text{MC}+w_{2}*\text{TC}. \end{array}$$

Equation (12) shows that the nodes with the smaller processing cost have the higher heuristic information amount and these nodes have more attraction for the ant *k*.

The pheromone amount *τ*_*uv*_ can reflect the attractiveness of the arc accessing to the destination node from the current node, which specifies how good the previous process plans are for the following ants. It will be updated according to the value of TPC of the process plan achieved by the ant *k*. The pheromone amount *τ*_*uv*_ can be given as follows:

(14) $$\begin{array}{c} \tau_{uv}^{k}=\left(1-\rho \right)*\tau_{uv}^{k}+\Delta \tau_{uv}^{k}, \end{array}$$

where  *ρ* is an evaporation coefficient of the pheromone on the arc linking the source node *u* and possible destination node *v*. Δ*τ*_*uv*_^*k*^ is the quantity of the pheromone trail on the arc(*u*, *v*) generated by the ant *k* after each iteration. Also, it can be given as

(15) $$\begin{array}{c} \Delta \tau_{uv}^{k}=\{\begin{array}{cc} \frac{Q}{\text{TPC}} & \text{if}\;\text{ant}\;k\;\text{passes}\;\text{the}\;\text{arc}\left(u,v\right),\\ 0 & \text{otherwise}, \end{array} \end{array}$$

where *Q* is a positive constant. Before ant colony begins the iteration, the pheromone amount on every arc is set to be *τ*_0_ initially.

The heuristic information and the pheromone amount constructed a probability of moving from a node to another node for an ant. The more the pheromone amount on the arc and the heuristic information on the node, the higher the selective probability. For the ant *k*, the selective probability *p*_*uv*_^*k*^ from the source node *u* to the destination node *v* can be given as follows:

(16) $$\begin{array}{c} p_{uv}^{k}=\{\begin{array}{cc} \frac{{\left[\tau_{uv}\right]}^{\alpha }{\left[\eta_{uv}^{k}\right]}^{\beta }}{\sum_{w\;\in \;S_{k}}{\left[\tau_{uw}\right]}^{\alpha }{\left[\eta_{uw}^{k}\right]}^{\beta }} & v\in S_{k},\\ 0 & v\notin S_{k}, \end{array} \end{array}$$

where *α* and *β* denote the weighting parameters controlling the relative importance of the pheromone amount and the heuristic information, respectively. *S*_*k*_ represents the set of nodes allowed to be visited at the next step for the ant *k*.

### 4.4. Termination

If all of the ants almost constructed the same process plans repeatedly at the early stage of the ACO algorithm, the algorithm would fall into the local convergence, which leads to failure in the exploration of new paths for the subsequent iteration. Once the algorithm falls into the local convergence, the output of process planning would not be the optimal result, even far from the optimal results. To void the local convergence, the parameter of *M*_*rpt*_ controlling the repeated number of the same process plan is set in advance. When the adjacent two-process plan is completely the same, the variable of *S*_*rpt*_ will increase by 1; otherwise *S*_*rpt*_ will be reset to be 0. When *S*_*rpt*_ reaches to *M*_*rpt*_, it means that no improvement on the solutions is made in the recent iterations. The ants may have converged to local optimal results. In addition, the local convergence occurs at the early stage of the ACO algorithm. To prevent the quick convergence, the maximum iteration *M*_*ite*_ is set in advance. Obviously, with the number of iterations *S*_*ite*_ increasing, even approaching to the *M*_*ite*_, the *M*_*rpt*_ will increase and can be calculated as follows:

(17) $$\begin{array}{c} M_{rpt}=S_{ite}*q*\frac{S_{ite}}{M_{ite}}, \end{array}$$

where *q* is random number, *q* ∈ (0, 1).

If the two events of *S*_*rpt*_ = *M*_*rpt*_ and *S*_*ite*_ < *M*_*ite*_ are satisfied simultaneously, it is considered that the local convergence occurs and the algorithm will be restarted. If the only event of *S*_*ite*_ = *M*_*ite*_ is satisfied, the resulting process plan will be output and algorithm will be terminated.

## 5. Experiments and Results

### 5.1. Walkthrough Example

When ACO is applied in process planning, those parameters including *K*, *ρ*, *α*, *β*, *E*, *Q*, *τ*_0_ have to be adjusted according to the situation to achieve the optimal process plan. The example part in Figure 2 is used to illustrate the proposed ACO approach. All the cost indexes are shown in Table 5 and it is assumed that all the machines and tools are available; namely, *w*_1_–*w*_5_ in (11) and (13) are set as 1.

A lot of preliminary experiments are dominated to test the effect of various parameters. In each experiment, one parameter is changed and the other parameters were fixed, and the effect of the changed parameter on the algorithm properties was analyzed at different levels. The resulting process plan is shown in Table 6 by the proposed ACO approach at the value of *K* = 5, *ρ* = 0.8, *α* = 2, *β* = 1, *E* = 45, *Q* = 1000, *τ*_0_ = 1, *M*_*ite*_ = 50.

### 5.2. Simulation Experiments

**Figure 4:**
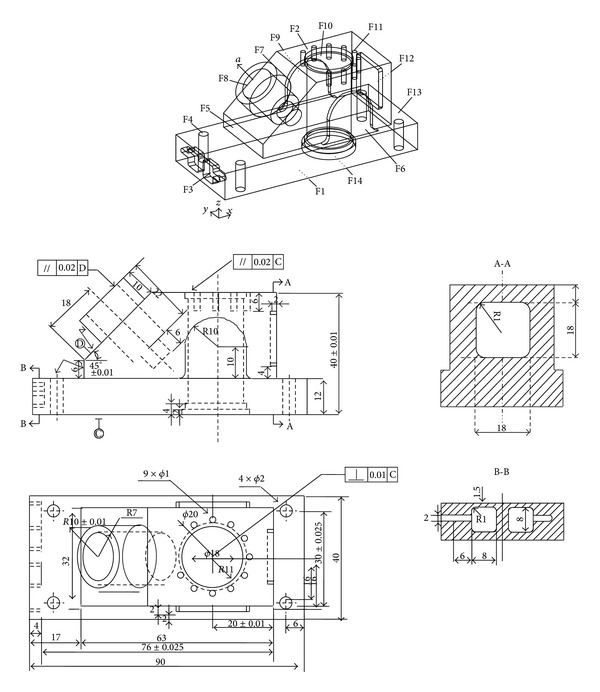
A sample part.

More complex process planning problems are considered in extensive simulation experiments. A sample part taken from the work of Li et al. [3, 4] is used to test the proposed ACO approach (Figure 4). The part consists of 14 defined manufacturing features, including planes, holes, and pockets. The detailed information of features, operations, manufacturing resources, and precedence relationship of the part is given in Tables 7, 8, and 9.

The above simulation experiment for the example part in Figure 2 shows that the selection of parameters is very important to the quality of the results. For the sample example in Figure 4, the method of determining those parameters is more complex, due to the enlargement of the problem size. It is assumed that all the machines and tools are available; namely, *w*_1_–*w*_5_ in (11) and (13) are set as 1.

The sample example is solved by the ACO approach with the varied values of *K* ∈ {5, 10, 20, 40}, *ρ* ∈ {0.05, 0.1, 0.25, 0.5, 0.8}, *α* ∈ {0.5, 1, 5, 10}, *β* ∈ {0.5, 1, 5, 10}, *E* ∈ {50, 55, 65, 80}, *Q* ∈ {1500, 2000, 2500, 3000}, and *M*_*ite*_ ∈ {100, 2000, 300, 400} and with the fixed value of *τ*_0_ = 1. 50 trials were separately conducted to evaluate the performance of the proposed approach. Experimental observation has shown that *K* = 10, *ρ* = 0.8, *α* = 1, *β* = 1, *E* = 80, *Q* = 3000, *τ*_0_ = 1, and *M*_*ite*_ = 200 are the best choices of these parameters. Four of the process plans generated are listed in Table 10. The best process plan (minimal TPC) is shown as process plan 1 in Table 10. The average result of 50 trials is shown in Table 11.

### 5.3. Comparative Tests

Three conditions are used to test the proposed algorithm for the sample parts [3, 4].

1. All machines and tools are available, and *w*_1_–*w*_5_ in (11) and (13) are set as 1.
2. All machines and tools are available, and *w*_2_ = *w*_5_ = 0, *w*_1_ = *w*_3_ = *w*_4_ = 1.
3. Machine M2 and tool T7 are down, *w*_2_ = *w*_5_ = 0, *w*_1_ = *w*_3_ = *w*_4_ = 1.

In Table 12, the TPC generated by the proposed ACO is compared with those of GA and SA approaches by Li et al. [3] and TS by Li et al. [4], as well as the ACO by Liu et al. [18].

Under condition (1), a lower TPC (2435.0) has been found using the proposed ACO approach, and the mean TPC (2456.1) is better than the costs of the other four algorithms. Under condition (2), the minimum TPC (2090) is the same as the ACO [6]. Under condition (3), the minimum TPC (2580) is the same as the TS [4]. The mean TPC generated by the proposed approach is better than the other four algorithms under the three conditions.

## 6. Conclusions

A graph-based ACO approach is developed to solve the process planning optimization problem against process constraints for prismatic parts, which considers the selection of machine resources, determining process operation, and sequencing operation according to machine cost. The approach is characterized by the following aspects.

(1) A graph-based representation of process plan is proposed. A weighted directed graph is used to represent process planning problem. The graph includes nodes set, directed arcs set, and undirected arcs set, which stand for operations, precedence constraints between the operations, and possible visited path connecting the nodes, respectively.

(2) A lower TPC is found by the proposed approach for the sample part, which means that the optimal process plan is generated by now under the same conditions. Comparing with the other algorithms, the proposed approach has generated the better process plan results under the three conditions.

In the further study, a deep discussion of selecting the ACO approach parameters is conducted. In addition, the multiobjective optimization will be incorporated into the ACO approach for handling the multiobjective process planning problem.

## Figures and Tables

**Table 1:** Operation selection for the example part.

Feathers	Operations	Machines	Tools	TADs
F1	Milling (Op_1_)	Vertical milling machine (M1)	Milling cutter (T1)	*+X,* +*Z *

F2	Drilling (Op_2_)	Vertical milling machine (M1)	Drill (T2)	*−Z *
	Tapping (Op_3_)	Drilling press (M2)	Tapping tool (T3)	

F3	Drilling (Op_4_)	Vertical milling machine (M1)	Drill (T4)	*−X *
	Reaming (Op_5_)	Drilling press (M2)	Reamer (T5)	

F4	Milling (Op_6_)	Vertical milling machine (M1)	Slot cutter (T6)	*+Z *

F5	Milling (Op_7_)	Vertical milling machine (M1)	Chamfer cutter (T7)	*−Z,* +*Y *

F6	Drilling (Op_8_)	Vertical milling machine (M1)	Drill (T8)	*+X *
	Reaming (Op_9_)	Drilling press (M2)	Reamer (T9)	

**Table 2:** Precedence constraints between operations.

Features	Operations	Precedence constraints description
F1	Op_1_	Op_1_ is prior to Op_2_ and Op_3_ for *Rule 2*. Op_1_ is prior to Op_4_ and Op_5_ for *Rule 5*.

F2	Op_2_	Op_2_ is prior to Op_3_ for *Rule 3*.

F3	Op_4_	Op_4_ is prior to Op_5_ for *Rule 3*.
	Op_4_, Op_5_	Op_4_ and Op_5_ are prior to Op_6_ for *Rule 5*. Op_4_ and Op_5_ are prior to Op_7_ for *Rule 5*.

F4	Op_6_	Op_6_ is prior to Op_2_ and Op_3_ for *Rule 4*.

F6	Op_8_	Op_8_ is prior to Op_9_ for *Rule 3*.
	Op_8_, Op_9_	Op_8_ and Op_8_ are prior to Op_7_ for *Rule 5*.

**Table 3:** Definition of a tool change.

Conditions of machining two consecutive operations	Tool change
Same tool and same machine	No
Same tool and different machines	Yes
Different tools and same machine	Yes
Different tools and different machines	Yes

**Table 4:** Definition of a setup change.

Conditions of machining two consecutive operations	Setup change
Same TAD and same machine	No
Same TAD and different machines	Yes
Different TADs and same machine	Yes
Different TADs and different machines	Yes

**Table 5:** Cost indexes for the example part in Figure 2.

MC		TC									MCC	TCC	SCC
M1	M2	T1	T2	T3	T4	T5	T6	T7	T8	T9			
40	10	10	3	7	3	8	10	10	3	8	300	60	20

**Table 6:** An optimal process plan for the example part in Figure 2.

Operation	Op_1_	Op_8_	Op_9_	Op_4_	Op_5_	Op_6_	Op_7_	Op_2_	Op_3_
Machine	M1	M1	M1	M1	M1	M1	M1	M1	M1
Tool	T1	T8	T9	T4	T5	T6	T7	T2	T3
TAD	*+X *	*+X *	*+X *	*−X *	*−X *	*+Z *	*−Z *	*−Z *	*−Z *

NMC = 0, NCC = 8, NSC = 4. TMC = 360, TTC = 62, TMCC = 0, TTCC = 480, TSCC = 80, TPC = 982.									

**Table 7:** Features, operations, and machining information of the sample part.

Features	Feature descriptions	Operations	TADs	Machines	Tools
F1	Planar surface	Milling (Op_1_)	+*Z*	M2, M3	T6, T7, T8

F2	Planar surface	Milling (Op_2_)	−*Z*	M2, M3	T6, T7, T8

F3	Two pockets arranged as a replicated feature	Milling (Op_3_)	+*X*	M2, M3	T6, T7, T8

F4	Four holes arranged as a replicated feature	Drilling (Op_4_)	+*Z*, −*Z*	M1, M2, M3	T2

F5	A step	Milling (Op_5_)	+*X*, −*Z*	M2, M3	T6, T7

F6	A protrusion (rib)	Milling (Op_6_)	+*Y*, −*Z*	M2, M3	T7, T8

F7	A boss	Milling (Op_7_)	−*a*	M2, M3	T7, T8

F8	A compound hole	Drilling (Op_8_)	−*a*	M1, M2, M3	T2, T3, T4
		Reaming (Op_9_)		M1, M2, M3	T9
		Boring (Op_10_)		M2, M3	T10

F9	A protrusion (rib)	Milling (Op_11_)	−*Y*, −*Z*	M2, M3	T7, T8

F10	A compound hole	Drilling (Op_12_)	−*Z*	M1, M2, M3	T2, T3, T4
		Reaming (Op_13_)		M1, M2, M3	T9
		Boring (Op_14_)		M3, M4	T10

F11	Nine holes arranged	Drilling (Op_15_)	−*Z*	M1, M2, M3	T1
		Tapping (Op_16_)		M1, M2, M3	T5

F12	A pocket	Milling (Op_17_)	−*X*	M2, M3	T7, T8

F13	A step	Milling (Op_18_)	−*X*, −*Z*	M2, M3	T6, T7

F14	A compound hole	Teaming (Op_19_)	+*Z*	M1, M2, M3	T9
		Boring (Op_20_)		M3, M4	T10

**Table 8:** Available machining resources and costs in a workshop environment.

Number	Types	MC
Machines		
M1	Drilling press	10
M2	Three-axis vertical milling machine	40
M3	CNC 3-axis vertical milling machine	100
M4	Boring machine	60

Number	Types	TC

Tools		
T1	Drill 1	7
T2	Drill 2	5
T3	Drill 3	3
T4	Drill 4	8
T5	Tapping tool	7
T6	Mill 1	10
T7	Mill 2	15
T8	Mill 2	30
T9	Ream	15
T10	Boring tool	20

MCC = 160, SCC = 100, TCC = 20		

**Table 9:** Precedence relationship between features and operations.

Features	Operation	Precedence constraints description
F1	Milling (Op_1_)	F1 (Op_1_) is the datum face for the part; hence, it is machined before all features

F2	Milling (Op_2_)	F2 (Op_2_) is before F10 (Op_12_, Op_13_, Op_14_) and F11 (Op_15_, Op_16_) for *Rule 2*

F3	Milling (Op_3_)	

F4	Drilling (Op_4_)	

F5	Milling (Op_5_)	F5 (Op_5_) is before F4 (Op_4_) and F7 (Op_7_) for *Rule 4*

F6	Milling (Op_6_)	F6 (Op_6_) is before F10 (Op_12_, Op_13_, Op_14_) for *Rule 4*

F7	Milling (Op_7_)	F7 (Op_7_) is before F8 (Op_8_, Op_9_, Op_10_) for *Rule 4*

F8	Drilling (Op_8_)	
	Reaming (Op_9_)	Op_8_ is before (Op_9_ and Op_10_); Op_9_ is before Op_10_ for *Rule 3*
	Boring (Op_10_)	

F9	Milling (Op_11_)	F9 (Op_11_) is before F10 (Op_12_, Op_13_, Op_14_) for *Rule 4*

F10	Drilling (Op_12_)	Op_12_ is before Op_13_ and Op_14_; Op_13_ is before Op_14_ for *Rule 3*; F10 (Op_12_, Op_13_, Op_14_) is before F11 (Op_15_, Op_16_) for *Rule 4*;Op_12_ of F10 is before F14 (Op_19_, Op_20_)
	Reaming (Op_13_)	
	Boring (Op_14_)	

F11	Drilling (Op_15_)	Op_15_ is before Op_16_ for *Rule 3*
	Tapping (Op_16_)	

F12	Milling (Op_17_)	

F13	Milling (Op_18_)	F13 (Op_18_) is before Op_4_ and Op_17_ for *Rule 2* and *Rule 1,* respectively

F14	Reaming (Op_19_)	Op_19_ is before Op_20_ for *Rule 3*
	Boring (Op_20_)	

**Table 10:** Four of the fifty process plans.

Process plan 1																				
Operation	1	2	18	11	6	12	13	19	17	3	5	7	8	9	10	20	14	4	15	16
Machine	2	2	2	2	2	2	2	2	2	2	2	2	2	2	2	4	4	1	1	1
Tool	7	7	7	7	7	3	9	9	7	7	7	7	3	9	10	10	10	2	1	5
TAD	+*Z*	−*Z*	−*Z*	−*Z*	−*Z*	−*Z*	−*Z*	+*Z*	−*X*	+*X*	+*X*	−*a*	−*a*	−*a*	−*a*	+*Z*	−*Z*	−*Z*	−*Z*	−*Z*

NMC = 2, NTC = 10, NSC = 9, TMCC = 320, TTCC = 200, TSCC = 900, TMC = 750, TTC = 265, TPC = 2435																				

Process plan 2																				
Operation	1	11	6	2	12	18	13	19	17	3	5	7	8	9	10	20	14	15	16	4
Machine	2	2	2	2	2	2	2	2	2	2	2	2	2	2	2	4	4	1	1	1
Tool	7	7	7	7	3	6	9	9	7	7	7	7	3	9	10	10	10	1	5	2
TAD	+*Z*	−*Z*	−*Z*	−*Z*	−*Z*	−*Z*	−*Z*	+*Z*	−*X*	+*X*	+*X*	−*a*	−*a*	−*a*	−*a*	+*Z*	−*Z*	−*Z*	−*Z*	−*Z*

NMC = 2, NTC = 11, NSC = 9, TMCC = 320, TTCC = 220, TSCC = 900, TMC = 750, TTC = 260, TPC = 2450																				

Process plan 3																				
Operation	1	5	3	18	6	2	11	12	13	17	7	8	9	19	14	20	10	4	15	16
Machine	2	2	2	2	2	2	2	2	2	2	2	2	2	2	4	4	4	1	1	1
Tool	6	6	6	6	6	6	7	3	9	7	7	2	9	9	10	10	10	2	1	5
TAD	+*Z*	+*X*	+*X*	−*Z*	−*Z*	−*Z*	−*Z*	−*Z*	−*Z*	−*X*	−*a*	−*a*	−*a*	+*Z*	−*Z*	+*Z*	−*a*	−*Z*	−*Z*	−*Z*

NMC = 2, NTC = 9, NSC = 10, TMCC = 320, TTCC = 200, TSCC = 1000, TMC = 770, TTC = 237, TPC =2527																				

Process plan 4																				
Operation	1	3	5	6	2	18	11	12	13	17	7	8	9	19	14	20	10	4	15	16
Machine	2	2	2	2	2	2	2	2	2	2	2	2	2	2	4	4	4	1	1	1
Tool	6	6	6	6	6	6	7	3	9	7	7	2	9	9	10	10	10	2	1	5
TAD	+*Z*	+*X*	+*X*	−*Z*	−*Z*	−*Z*	−*Z*	−*Z*	−*Z*	−*X*	−*a*	−*a*	−*a*	+*Z*	−*Z*	+*Z*	−*a*	−*Z*	−*Z*	−*Z*

NMC = 2, NTC = 9, NSC = 10, TMCC = 320, TTCC = 200, TSCC = 1000, TMC = 770, TTC = 237, TPC = 2527																				

**Table 11:** Average results of simulation experiment.

Type	Mean	Maximum	Minimum	Standard deviation
TMC	754.2	800	750	9.82
TTC	261.88	267	237	7.63
TMCC	320	320	320	320
TTCC	202	220	180	10.77
TSCC	918	1000	900	38.42
TPC	2456.1	2527.0	2435.0	37.98

**Table 12:** Results compared to other algorithms for the sample part in Figure 4.

Condition	Proposed approach	ACO	TS	SA	GA
(1)					
Mean	2456.1	2490.0	2609.6	2668.5	2796.0
Maximum	2527.0	2500.0	2690.0	2829.0	2885.0
Minimum	2435.0	2450.0	2527.0	2535.0	2667.0
(2)					
Mean	2115.4	2117.0	2208.0	2287.0	2370.0
Maximum	2380.0	2120.0	2390.0	2380.0	2580.0
Minimum	2090.0	2090.0	2120.0	2120.0	2220.0
(3)					
Mean	2600	2600.0	2630.0	2630.0	2705.0
Maximum	2740.0	2600.0	2740.0	2740.0	2840.0
Minimum	2580.0	2600.0	2580.0	2590.0	2600.0
